# Thio Analogs of Pyrimidine Bases: Synthesis, Spectroscopic Study, and *In Silico* Biological Activity Evaluation of New 2-*o*-(*m*- and *p*-)Chlorobenzylthio-6-Methyl-5-Piperidino-(Morpholino-)Methyluracils

**DOI:** 10.5402/2011/610521

**Published:** 2011-04-07

**Authors:** Tomasz Pospieszny, Marcin Szymankiewicz, Elżbieta Wyrzykiewicz

**Affiliations:** Faculty of Chemistry, Adam Mickiewicz University in Poznań, Grunwaldzka 6, 60-780 Poznań, Poland

## Abstract

Six new 2-*o*-(*m*- and *p*-)chlorobenzylthio-6-methyl-5-piperidino-(or morpholino-) methyluracils have been prepared. The structures of these compounds were confirmed by spectroscopic (FT-IR, UV-Vis, ^1^H NMR, ^13^C NMR, and HMBC) and elemental analyses. Estimation of pharmacotherapeutic potential has been made for synthesized compounds on the basis of prediction of activity spectra for substances (PASS).

## 1. Introduction

Thio derivatives of pyrimidine bases have remarkably contributed to biological and medicinal chemistry. Chemical modification of these compounds have led to a large number of mono- and di-*S* and *N*-substituted analogs showing therapeutic properties, especially antivirial, antithyroid, and antitumor activities [1–7]. The antimetabolites of 5,6-dimethyluracil 5-morpholinomethyl-6-methyl-2-thiouracil [8] and 5-piperidinomethyl-6-methyl-2-thiouracil [9] have been synthesized via the Mannich reaction. However, to the best of our knowledge no work has been published on the synthesis as well as on physicochemical properties of the monochlorobenzylthio-substituted derivatives of these compounds. This fact has stimulated us to investigate the reaction of chlorobenzylation of 2-thio-5-piperidinomethyl-6-methylthiouracil and 2-thio-5-morpholinomethyl-6-methylthiouracil as well as the Mannich reaction of 2-*o*-(*m*- and *p*-)chlorobenzylthio-6-methylthiouracils, formaldehyde, and piperidine (or morpholine). Recently novel pharmacological action of 2,4-di-*o*-(*m*- and *p*-)bromo-(chloro- and nitro-)benzylthio-5-bromouracils (and 6-methyluracils) [10] as well as disulfides of *N*,*O*-(*N*,*N*- or *O*,*O*-)-di- and *N*,*N*,*O*-tri-(*o*-, *m*- and *p*-)-bromobenzyl-2-thiouracils [11] has been found on the basis of the computer-aided drug discovery approach with the compounds program Prediction of Activity Spectra for Substances (PASS) [12–15]. Since only the structural formula of the chemical compound is necessary to obtain a PASS prediction, this approach was used in the present work. This paper deals with the synthesis and physicochemical properties of **1–6**. Additionally, the analysis of biological activity spectra prediction for **1–6** made in this paper is a good example of *in silico* studies of chemical compounds.

## 2. Results and Discussion

**Scheme 1:**
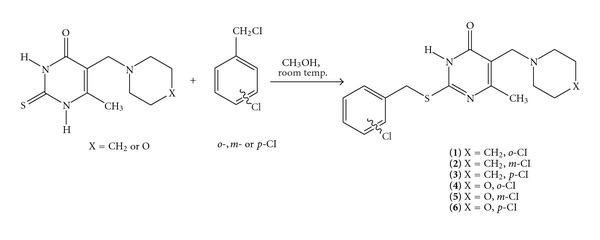


**Scheme 2:**
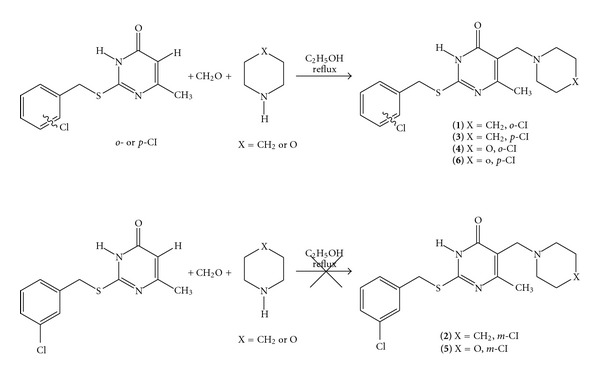


A series of six new 2-*o*-(*m*- and *p*-)chlorobenzylthio-5-piperidinomethyl-6-methyluracils **1–3** and 2-*o*-(*m*- and *p*-)chlorobenzylthio-5-morpholinomethyl-6-methyluracils **4–6 **were synthesized by the reaction of 2-thio-5-piperidinomethyl-6-methyluracil or 2-thio-5-morpholinomethyl-6-methyluracil in 3 N NaOH in methanol with *o*-(*m*- and *p*-)chlorobenzyl chlorides at room temperature for 24 h (Scheme 1). Compounds **1**, **3**, **4**, and **6** were also produced in well-known Mannich reaction [16] from 2-*o*-(and *p*-)chlorobenzylthio-6-methyluracils [17] when refluxed with paraformaldehyde and piperidine (or morpholine) in ethanol (Scheme 2). Compounds **2** and **5** were not produced in the Mannich reaction of relatively soft 2-*m*-chlorobenzylthio-6-methyluracils with paraformaldehyde and piperidine (or morpholine) when heated in ethanol (Scheme 2). The 2 *o*-(*m*- and *p*-)chlorobenzylthio-containing compounds **1–6** were confirmed by examination of their UV/Vis, FT-IR, ^1^H NMR and ^13^C NMR spectra (Tables 1 and 2), and HMBC (Table 3) as well as elemental analyses (Table 4).

The ^1^H NMR and ^13^C NMR data of **1–6** are given in Tables 1 and 2, respectively. Assignments of the ^1^H NMR and ^13^C NMR resonances of these compounds were deduced on the basis of the signal multiplicities and by the corrected application of two-dimensional NMR technique ^1^H-^1^H COSY and HMBC. The ^1^H NMR spectra of **1–6** reveal singlets of S–CH_2_ at 4.29–4.46 ppm. The singlets of C_5_–CH_2_ of **1–6** are situated at 3.28–3.58 ppm, and the singlets of C_6_–CH_3_ of **1–6** are situated at 2.22–2.33 ppm, respectively.

The ^13^CNMR spectra of compounds **1–6** in DMSO*_d6_* showed characteristic signals in the range of 31.55–32.81 ppm and 53.04–53.71 ppm assigned to S–CH_2_ and N–CH_2_, respectively. The thiouracil ring exhibited signals in the range 110.01–114.71 ppm and 158.81–162.11 ppm assigned to C_5_=C_6_, respectively. The ^13^C NMR spectra of compounds **1–6** showed the presence of a methyl group from the 6-methyl-2-thiouracil ring at 20.90–21.30 ppm.

The HMBC spectrum clearly shows the connectivities of all hydrogen and carbon atoms involved, including quaternary carbons. The HMBC results allow an unequivocal assignment of *S*-substitution of benzyl group at uracil ring of **1–6 **(Table 3). The HMBC experiment is conducted without ^13^C decoupling so that correlations via one or more bond can be discerned and one-bond correlation affords double cross-peaks in the ^1^H dimension. For compounds **1–6** the ^1^H NMR spectrum exhibits three singlets at 4.29–4.46, 3.28–3.58, and 2.22–2.33 ppm ascribed to protons of S–CH_2_, C_5_–CH_2_ and C_6_–CH_3_, respectively. The HMBC of **1–6** shows peaks corresponding to two-bond correlations for C_6_–CH_3_/C_6_ (158.81–162.11 ppm) and three-bond correlations for S–CH_2_/C_2 _(163.68–168.05 ppm) and C_5_–CH_2_/C_4_ (161.82–163.19 ppm).

The FT-IR spectra of **1–6** show absorption bands of medium intensities in the region 809–820 cm^−1^ assigned to **ν** C–Cl vibration as well as in the region 1035–1091 cm^−1^ assigned to **δ** C–Cl vibration (Table 1). The FT-IR spectra of **1–6** show also absorption bands in the region 2852–2864 cm^−1^ assigned to **ν** CH_2_–S as well as in the region 1444–1447 cm^−1^ assigned to **δ** CH_2_–S vibration (Table 1). The UV/Vis spectra of **1–6** show *λ*_max_ in the range 245–250 nm (Table 1).

In the present paper the biological activity spectra were predicted for all six synthesized compounds (**1–6**) using PASS [12–15]. We have also selected the types of activities that were predicted for a potential compound with the highest probability (focal activities). They are presented in Table 5. According to these data the most frequently predicted types of biological activities are antiviral (Influenza), antiseborrheic, and prolyl aminopeptidase inhibitor. It ought to be pointed out that in the series of compounds **1**, **3** and **6** such activity as mucomembranous protector has also been predicted, as well as in the series of compounds **2**, **3**, **5,** and **6** such activity as prolyl aminopeptidase inhibitor.

## 3. Conclusions

The reaction of 2-thio-5-piperidinomethyl-6-methyluracil and 2-thio-5-morpholinomethyl-6-methyluracil with *o*-(*m*- and *p*-)chlorobenzyl chlorides in 3 N NaOH in methanol at room temperature leads to 2-*o*-(*m*- and *p*-)chlorobenzylthio-5-piperidinomethyl-6-methyluracils **1–3** and 2-*o*-(*m*- and *p*-)-chlorobenzylthio-5-morpholinomethyl-6-methyluracils **4–6**. The Mannich reaction of 2-*o*-(and *p*-)chlorobenzylthio-6-methyluracils with paraformaldehyde and piperidine (or morpholine) refluxed in ethanol leads to **1**, **3**, **4**, and **6**. The results obtained by PASS method of identification of the prospective pharmacological properties of **1–6** exhibit the possibility of finding new pharmacological agents from this class of compounds. 

## 4. Experiment

The purity of all described compounds was checked by melting points, TLC, and elemental analyses. Melting points (uncorrected) were determined on a Boetius microscope hot stage. Rf values refer to silica gel F_254_ TLC plates (Merck) developed with CHCl_3_ : CH_3_OH (10 : 1) and observed under UV light (*λ* = 254 and 366 nm). UV/Vis spectra were recorded with a SPECORD UV/Vis Spectrophotometer in methanol. IR spectra were recorded with FT-IR Bruker IFS-113 Spectrophotometer in KBr pellets. The ^1^H NMR (300 MHz) and ^13^C NMR (75 MHz) spectra were determined with Varian Gemini 300 spectrometer in DMSO*_d6_* solution at a concentration between 0.25 and 0.40 M in the 5 mm sample tubes at ambient temperature. Chemical shifts are given in *δ* scale (ppm). Elemental analyses were performed with a Vector Euro EA 3000 analyzer. 

2-thio-5-piperidinomethyl-6-methyluracil [9], 2-thio-5-morpholinomethyl-6-methyl-uracil [8], and 2-*o*-(*m*- and *p*-)-chlorobenzylthio-6-methyluracils [17] have been obtained according to the literature.

The synthesis of 2-*o*-(and *p*-)chlorobenzylthio-5-piperidinomethyl-6-methyluracils (**1** and **3**) and 2-*o*-(and *p*-)chlorobenzylthio-5-morpholinomethyl-6-methyluracils (**4 **and** 6**) is as follows. 

General procedure A: 0.2 g (0.8 mmole) of 2-thio-5-piperidino-(morpholino-)-methyl-6-methyluracils [8, 9] was dissolved with stirring in room temperature in 2.5 mL of 3 N NaOH in methanol. Next, to the solution 0.116 mL (0.9 mmole) of *o*-chlorobenzyl chloride or 0.146 g (0.9 mmole) of *p*-chlorobenzyl chloride was added. After stirring at room temperature for 24 hours, the obtained crude product was filtered off and crystallized from methanol.

The synthesis of 2-*m*-chlorobenzylthio-5-piperidinomethyl-6-methyluracils (**2**) and 2-*m*-chlorobenzylthio-5-morpholinomethyl-6-methyluracils (**5**) is as follows. 

General procedure A: 0.2 g (0.8 mmole) of 2-thio-5-piperidino-(morpholino-)methyl-6-methyluracils [8, 9] was dissolved with stirring in room temperature in 2.5 mL of 3 N NaOH in methanol. Next, to the solution 0.116 mL (0.9 mmole) of *m*-chlorobenzyl chloride was added. The reaction mixture was stirred in room temperature for 24 hours and after that time 5 mL distilled water was added. The obtained crude product was collected by filtration, washed with distilled water, and dried in the exicator. The obtained dry solid was dissolved in 10 mL of CHCl_3_ and separated by silica gel column chromatography (Merck 203–400 mesh) using the following solvent mixtures: CHCl_3_ : CH_3_OH 50 : 1 (40 mL), 40 : 1 (40 mL), 30 : 1 (30 mL), 20 : 1 (30 mL), and 10 : 1 (20 mL). The fractions of 20 mL were collected. On the basis of analytical TLC fractions of product desired were obtained by combining 20 mL fractions. They were concentrated on a rotary evaporator. Compounds **2** and **5** were shown to by analytically pure.

The synthesis of 2-*o*-(and *p*-)chlorobenzylthio-5-piperidinomethyl-6-methyluracils (**1** and **3**) and 2-*o*-(and *p*-)chlorobenzylthio-5-morpholinomethyl-6-methyluracils (**4 **and** 6**) is as follows. 

General procedure B: A mixture of 0.1 g (0.38 mmole) 2-*o*-(or *p*-)chlorobenzylthio-6-methyluracils, 0.024 g (0.4 mmole) paraformaldehyde, and 0.0035 mL (0.4 mmole) morpholine (or 0.0046 mL, 0.4 mmole piperidine) was suspended in 6 mL of ethanol (99.8%) and refluxed for 8 hours. The precipitated solid was isolated by filtration, dried in room temperature, and recrystallized from methanol.

## Figures and Tables

**Table 1:** FT-IR, UV-Vis, and ^1^H NMR data of compounds **1–6**.

Comp.	UV/Vis (CH_3_OH) *λ*_max_ nm (log *ε*)	FT-IR cm^−1^(KBr)		^1^H NMR (DMSO*_d6_*) ppm		
		S–CH_2 _(**ν**)	C–Cl (**ν**)	S–CH_2_ (s)	C_5_–CH_2_ (s)	C_6_–CH_3_ (s)
		S–CH_2 _(*δ*)	C–Cl (*δ*)			
**1**	250.0 (3.03)	2857.5	1052.5	4.39	3.58	2.23
		1447.4	820.3			
**2**	250.0 (3.00)	2856.8	1035.0	4.30	3.58	2.22
		1446.3	822.1			
**3**	249.5 (2.87)	2864.6	1055.3	4.29	3.57	2.22
		1450.9	819.6			
**4**	247.0 (3.27)	2852.5	1053.1	4.46	3.29	2.33
		1444.0	814.1			
**5**	245.5 (3.34)	2858.5	1059.6	4.36	3.29	2.31
		1456.3	813.0			
**6**	247.5 (3.23)	2858.8	1091.3	4.35	3.28	2.31
		1444.7	809.6			

**Table 2:** ^13^C NMR data of compounds **1–6**.

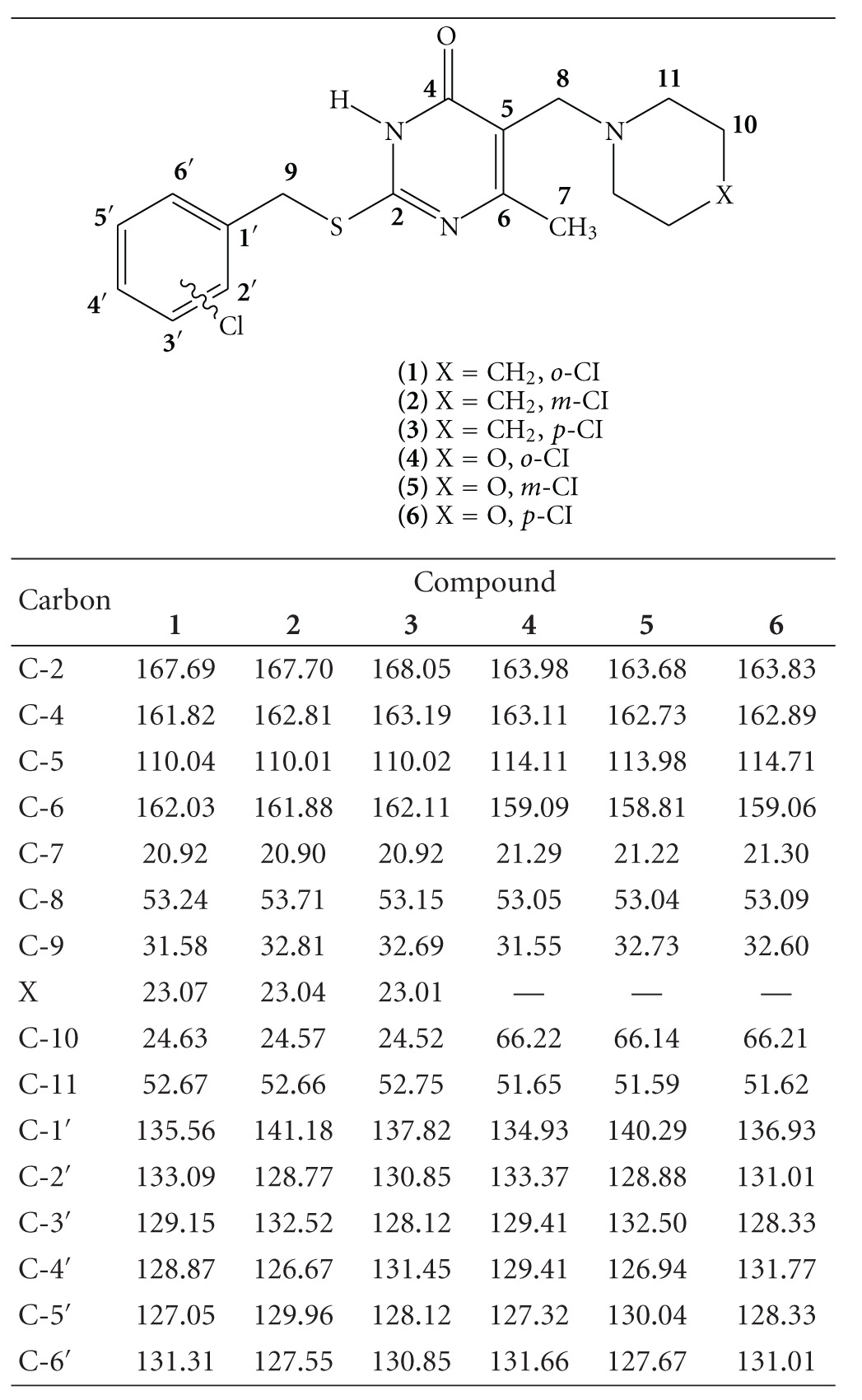

**Table 3:** The HMBC correlations between protons and carbons of compounds **1–6**.

Compound	Carbon atom	*δ*C (ppm)	HMBC ^1^H/^13^C
**1**	C-2	167.69	S–CH_2_
	C-4	161.82	C_5_–CH_2_
	C-6	162.03	C_6_-CH_3_

**2**	C-2	167.70	S–CH_2_
	C-4	162.81	C_5_–CH_2_
	C-6	161.88	C_6_-CH_3_

**3**	C-2	168.05	S–CH_2_
	C-4	163.19	C_5_–CH_2_
	C-6	162.11	C_6_–CH_3_

**4**	C-2	163.98	S–CH_2_
	C-4	163.11	C_5_–CH_2_
	C-6	159.09	C_6_–CH_3_

**5**	C-2	163.68	S–CH_2_
	C-4	162.73	C_5_–CH_2_
	C-6	158.81	C_6_–CH_3_

**6**	C-2	163.83	S–CH_2_
	C-4	162.89	C_5_–CH_2_
	C-6	159.06	C_6_–CH_3_

**Table 4:** Physical and analytical data of compounds **1–6**.

Comp.	Formula MW	Yield [%]	M.p. [°C]	Rf TLC*	Analysis					
					Calculated			Found		
					C	H	N	C	H	N
**1**	C_18_H_22_N_3_SOCl 363.90	58	183-3	0.21	59.12	5.89	11.55	59.00	5.60	11.40
**2**	C_18_H_22_N_3_SOCl 363.90	35	147-8	0.20	59.12	5.89	11.55	59.59	5.72	11.60
**3**	C_18_H_22_N_3_SOCl 363.90	67	99-100	0.23	59.12	5.89	11.55	59.10	5.80	11.28
**4**	C_17_H_20_N_3_SO_2_Cl 365.88	62	175-6	0.30	55.81	5.51	11.48	55.53	5.24	11.21
**5**	C_17_H_20_N_3_SO_2_Cl 365.88	23	134-5	0.31	55.81	5.51	11.48	55.82	5.34	11.16
**6**	C_17_H_20_N_3_SO_2_Cl 365.88	56	177-8	0.32	55.81	5.51	11.48	55.58	5.30	11.24

*CHCl_3_ : CH_3_OH, 10 : 1.

**Table 5:** PA values for predicted biological activity of compounds **1–6**.

Compound	Focal predicted activity (PA > 0.7)
**1**	Mucomembranous protector (0.777)
	Antiviral (Influenza) (0.711)

**2**	Cardiotonic (0.790)
	Antiviral (Influenza) (0.704)
	Antiseborrheic (0.768)
	Prolyl aminopeptidase inhibitor (0.751)

**3**	Mucomembranous protector (0.796)
	Antiviral (Influenza) (0.711)
	Antiseborrheic (0.780)
	Prolyl aminopeptidase inhibitor (0.720)

**4**	Antiviral (Influenza) (0.721)

**5**	Antiviral (Influenza) (0.711)
	Antiseborrheic (0.768)
	Prolyl aminopeptidase inhibitor (0.735)

**6**	Mucomembranous protector (0.712)
	Antiviral (Influenza) (0.722)
	Antiseborrheic (0.778)
	Prolyl aminopeptidase inhibitor (0.735)
